# Ability to Suppress TGF-β-Activated Myofibroblast Differentiation Distinguishes the Anti-pulmonary Fibrosis Efficacy of Two Danshen-Containing Chinese Herbal Medicine Prescriptions

**DOI:** 10.3389/fphar.2019.00412

**Published:** 2019-04-24

**Authors:** Rui Shao, Fu-jiang Wang, Ming Lyu, Jian Yang, Peng Zhang, Yan Zhu

**Affiliations:** ^1^Tianjin State Key Laboratory of Modern Chinese Medicine, Tianjin University of Traditional Chinese Medicine, Tianjin, China; ^2^Research and Development Center of Tianjin University of Traditional Chinese Medicine, Tianjin International Joint Academy of Biotechnology & Medicine, Tianjin, China

**Keywords:** idiopathic pulmonary fibrosis, pulmonary-heart disease, Danlou prescription, Danhong prescription, myofibroblast differentiation

## Abstract

**Background:** Idiopathic pulmonary fibrosis (IPF) is a devastating lung disease with limited treatment options. It also leads to progressive respiratory failure, which subsequently affects the heart functionality, a pathological heart-lung interaction increasingly noticed and defined as pulmonary-heart disease (PHD). Traditional Chinese medicine (TCM) theory for treating “phlegm-stasis cementation syndrome” may suggest a possibility of treating PHD complication with Chinese medicine prescriptions previously used for cardiovascular diseases.

**Methods:** Here, we evaluate the efficacies of two compound Chinese medicine prescriptions, Danlou prescription (DLP) and Danhong prescription (DHP), which share a common herbal component, Salvia miltiorrhiza (Danshen), on pulmonary fibrosis. Severity grades of Bleomycin (BLM)-induced pulmonary fibrosis were assessed by micro-Computerized Tomography (μCT) in accordance with the clinical evaluation standard. Lung pathological changes and collagen deposition were investigated by histopathology. Myofibroblast differentiation was assessed by immunohistochemistry of α-SMA and TGF-β receptor type II expression *in situ*. Network pharmacology analysis of the drug-target interaction in IPF progression for DLP or DHP was performed using Ingenuity^®^ Pathways Analysis (IPA) system.

**Results:** We show that a non-invasive μCT effectively monitor and quantify BLM-induced pulmonary fibrosis and its treatment efficacy by Chinese medicine prescription in rodents. In addition, although both containing Salvia miltiorrhiza, DLP but not DHP mitigates BLM-induced lung fibrosis by inhibiting the TGF-β signaling-activated myofibroblast differentiation and α-SMA expression in a mouse model. Core analysis by IPA revealed that DLP ingredients regulated not only pulmonary fibrosis related inflammatory genes but also genes associated with myofibroblast activation and collagen deposition.

**Conclusion:** This study suggests that a clinically efficacious cardiovascular Chinese herbal medicine (DLP) can be successfully repurposed to treat a lung disease in pulmonary fibrosis guided by TCM theory. Our comparative study between DLP and DHP demonstrated a critical requirement of suppressing both pro-inflammatory and pro-fibrotic pathways for the treatment of pulmonary fibrosis, supporting that a multi-component prescription capable of “removing both phlegm and blood stasis” will better achieve co-protection of heart and lung in PHD.

## Introduction

Idiopathic pulmonary fibrosis (IPF) is a chronic and life-threatening disease characterized by a progressive decline in lung function (Gross and Hunninghake, 2001). It is associated with an extremely poor prognosis and limited curative therapies (Raghu et al., 2011; Richeldi et al., 2017), causing the median survival time of patient from diagnosis to 2–4 years and very high lethality (Bjoraker et al., 1998; Ley et al., 2011; Richeldi et al., 2017). The pathogenesis of IPF was hypothesized to be due to the excessive deposition of dysregulated extracellular matrix (ECM) proteins leading to fibroblast proliferation and abnormal re-epithelialization, following alveolar injury and progressive respiratory failure (Wynn and Ramalingam, 2012). Since varieties of chronic respiratory diseases may be accompanied with the remodeling of right ventricle (Fishman, 1976), abnormal lung function also can affect the heart functionality (Billingham, 1995; Yuki et al., 2009; Rozova and Mankovska, 2012). Therefore, pulmonary heart disease (PHD) is named for the consideration of heart and lung as one function-coupled unit (Forfia et al., 2013).

Considering the drug candidates that have been assessed or are under assessment for IPF (Wuyts et al., 2014), it was striking that only few molecules were shown to reduce disease progression of IPF except pirfenidone and nintedanib (King et al., 2014; Richeldi et al., 2014). With limited treatment options available, combination therapy has been suggested as the future of management for IPF (Wuyts et al., 2014). As a natural combination medicine, compound Chinese herbal medicine holds a great promise for IPF and PHD. One fundamental principal of traditional Chinese medicine (TCM) is syndrome-targeted compatibility of different herbal components. However, the differential efficacies and underlying mechanisms of the “prescription compatibility” remain incompletely illustrated.

Two clinically used cardiovascular Chinese medicines, DanLou prescription (DLP) and DanHong prescription (DHP) (Zhi et al., 2012; Wang et al., 2015; Qi et al., 2016; Yang et al., 2016), which share a common herbal component, Salvia miltiorrhiza (Danshen), to validate the efficacy and mechanism in pulmonary fibrosis model. DLP is developed from the TCM formula Gualou Xiebai decoction and is composed of 10 herbs: Trichosanthes kirilowii Maxim. (Gualoupi), Allium macrostemon Bge. (Xiebai), Pueraria lobata Ohwi (Gegen), Salvia miltiorrhiza Bge. (Danshen), Astragalus membranaceus Bge. var. mongholicus Hsiao (Huangqi), Alisma orientalis Juzep. (Zexie), Drynaria fortune (Kunze) J. Sm. (Gusuibu), Ligusticum chuanxiong Hort. (Chuanxiong), Paeonia lactiflora Pall. (Chishao), and Curcuma longa L. (Yujin) (Dong J. et al., 2013). DLP inhibits foam cell formation induced by ox-LDL via the TLR4/NF-κB and PPARγ signaling pathways (Gao et al., 2018). Gualou Xiebai decoction, a classic form of DLP, prevents cardiac reperfusion injury of hyperlipidemia rat via regulating energy metabolism involving inactivation of RhoA/ROCK signaling pathway (Yan et al., 2018). DHP contains only two herbs: Radix *Salviae miltiorrhizae* (Danshen) and *Flos Carthami tinctorii* (Honghua). According to TCM theory, both DLP and DHP are capable of “promoting circulation and resolving blood stasis,” an essential feature to treat “blood stasis syndrome.” However, DLP is better suited for “phlegm-stasis cementation syndrome” with its ability to “removing both phlegm and blood stasis.”

Bleomycin (BLM)-induced pulmonary fibrosis is a well-established IPF model to study the efficacy and mechanism of therapeutic candidates (Song et al., 2010; Chen et al., 2013). Direct instillation of BLM into the airway may cause injury to the lung epithelium and endothelium and elicit inflammatory responses (Haslett et al., 1989; Sonoda et al., 2014), which reproduce typical features of the human disease (Usuki and Fukuda, 1995). There are three stages of IPF in a mouse model beginning with an acute inflammatory stage, involving alveolar epithelial cell damage, inflammatory cell recruitment, and pro-inflammatory mediator release. A sub-acute stage follows, with pro-fibrotic cytokine expression and fibroblast proliferation and differentiation around the sites of injury. A final stage is characterized by increased collagen deposition and fibrosis (Iyer et al., 2000; Williamson et al., 2015). Transforming growth factor (TGF)-β1, a pro-fibrotic cytokine, interacts with a heteromeric complex of transmembrane serine/threonine kinase receptors, containing type I (TβRI) and the type II (TβRII) receptors (Bataller and Brenner, 2005). Earlier study demonstrated that TβRII promoted fibroblast differentiation, resulting in resistance to BLM-induced pulmonary lesions in mice (Li et al., 2011; Mouw et al., 2014). Since a hallmark of myofibroblast differentiation is the expression of alpha-smooth muscle actin (α-SMA), an underlying mechanism of pulmonary fibrosis could be assessed by the expression levels of α-SMA and TβRII.

Here, we are interested in whether these two different cardiovascular medicines have effects on the blood stasis in lung fibrosis sites. To date, we presented the morphology of fibrosis tissue by μCT scanning (Choi et al., 2014), together with histopathological assay and immunohistochemistry assay, in order to test DLP and DHP therapy effects, laying a solid foundation for developing cardiovascular and IPF combination therapy based on TCM prescription compatibility theory.

## Materials and Methods

### Chemicals and Reagents

DanLou Tablet (Batch Number: 20140101038, 0.3g/tablet), a commercial preparation of DLP, was obtained from Jilin Kangnaier Pharmaceutical Group Co. Ltd. (Jilin, China). The chromatograms with characterization of the dominating compound (s) of DanLou Tablet was determined as same as those shown in our previous studies (Dong J. et al., 2013a; Wu et al., 2014). DanHong injection (Batch number: 12081024077, 10 ml/ampulla), a commercial preparation of DHP, comprising 750 g Salvia miltiorrhiza, 250 g Safflower, and 7 g Sodium chloride was supplied by Heze Buchang Pharmaceutical Co., Ltd (Shandong, China). The chromatograms with characterization of the dominating compound (s) of DanHong injection was determined as same as those shown in our previous studies (Liu et al., 2013). Bleomycin (BLM) hydrochloride was purchased from Nippon Kayaku Co. (Tokyo, Japan). Rosiglitazone (ROS) (Batch number: 122320-73-4), which has been well established by invasive techniques to reduce lung fibrosis (Burgess et al., 2005; Lee et al., 2008; Jin et al., 2012), was purchased from Alexis Biochemicals (San Diego, CA, United States). Mouse anti-α-SMA antibody was purchased from Boster Biotec (Wuhan, China). Rabbit anti-TβRII antibody was purchased from Santa Cruz Biotechnology (Santa Cruz, CA, United States). Chloral hydrate (Batch number: Q/12HB 4218-2009) was purchased from Tianjin Kermel Chemical Reagent Co. Ltd. (Tianjin, China), freshly prepared to 3.5% solution with saline before experiment. HE Staining Kit was purchased from Wuhan Boster Biological Co., Ltd. (Wuhan, China). Formalin was purchased from Shanghai Weiao Biological Co., Ltd. (Shanghai, China). Sodium chloride injection was purchased from Cisen Pharmaceutical Co., Ltd. (Shandong, China).

### Animals Experimental Protocols

Seventy male C57BL/6 mice, weighing 20–25 g, were purchased from HFK Bioscience Co, Ltd. (Beijing, China) and housed in a 12 h light/dark cycled facility with free access to food and water. All experiments were reviewed and approved by the Institutional Animal Care and Use Committee at the Tianjin International Joint Academy of Biotechnology and Medicine (TJAB-JY-2011-002) and were carried out under the Guidelines for Animal Experiments of Tianjin University of TCM.

IPF was established in mice model by intratracheal instillation of BLM hydrochloride (5 mg/kg dissolved in 0.9% saline). After the bleomycin treatment, mice were frequently examined for 2 weeks by μCT. Mice presenting fibrosis lesions involving more than 25% of the lung (Model group) were divided into three experiment groups, treating once daily with DLP, DHP and Rosiglitazone (ROS, the positive control drug), respectively, for another 2 weeks. DLP and ROS were administered via oral gavage and DHP was administered via intraperitoneal injection. According to the normal clinical analysis (Guo et al., 2014; Yao et al., 2017), the mouse equivalent dose of DLP and DHP were calculated on the basis of body surface area by multiplying the human dose (43 mg/kg and 0.20 ml/kg body weight) by the correction factor ratio (12.3) (Nair and Jacob, 2016). This calculation resulted in a mouse equivalent dose for DLP and DHP of 530 mg/kg and 2.5 ml/kg body weight, which set to be the medium drug concentration in our study. Thus, we set up three gradient concentrations for DLP (177, 530, 1590 mg/kg body weight) and DHP (0.8, 2.5, 7.5 ml/kg body weight), and ROS (5 mg/kg bodyweight) to treat the BLM-induced pulmonary fibrosis mice. Micro-CT scans were performed at baseline, 2 and 4 weeks to evaluate the effect of these treatments. Mice without treatments (Normal group), treated with BLM alone but no fibrosis or inflammation findings (Sham group) were given an equal volume of saline in the same schedule.

Mice were sacrificed at 4 week and the lower lobes of left lung tissues were taken and fixed by 10% formalin. The mice lung imaging was performed using a μCT scanner (IvIS Lumina K series III, PerkinElmer) for 5 min with help of computer software (Living Image version 4.3.1, PerkinElmer). The X-ray system uses a microfocus tube with a spot size of 5 μm and produces X-rays in a cone-beam geometric formation. The images were acquired at 90 kV, 160 mA and the wide field of view (FOV) scanning at 40 mm. The image field width was up to 68 mm and the voxel size was 35 × 35 × 35 μm. The pathological grade of inflammation and fibrosis in the whole lung was evaluated under 25–100 magnifications and determined according to the criteria. Moreover, the Wilcoxon signed-rank test using statistical methods was conducted to compare the μCT images of different groups after 2 and 4 weeks (Natarajan et al., 2012). Abnormal areas on μCT imaging were evaluated before and after ROS administration. Observes gave a point (from 0 to 5 scores) at each level for consolidation, GGA, reticular opacity and honeycombing according to method proposed by Lee et al. (2008).

### Histopathological Assay

The lower lobes of left lung tissues were fixed in 10% formalin, following embedded in paraffin wax. After that 5 μm sections of lung edge were cut and selected for hematoxylin and eosin (H&E) stain, hematoxylin colors nuclei of cells (and a few other objects, such as keratohyalin granules and calcified material) blue. The nuclear staining was followed by counterstaining with an aqueous solution of eosin, which colors eosinophilic structures in various shades of pink.

In the process of Masson’s trichrome stain, it produces green collagen, pink cytoplasm and dark brown of cell nuclei. The fibrosis results of Masson’s trichrome stain were graded according to Modified Ashcroft Scale (Hubner et al., 2008), which were quantified from grade 0 to 8 designed for a standardized fibrosis evaluation in small animals, with higher score corresponding to a more serious level of fibrosis. As an indirect parameter of fibrosis, the alveolar air area was quantified by Image-Pro Plus 6.0 software (Media Cybernetics; Rockville, MD, United States). The Air Area is expressed as a percentage of area (μm^2^) occupied by air referred to the lung total area (μm^2^) within the region of interest (ROI). Bronchi and blood vessels have been removed from the ROI area.

### Immunohistochemistry (IHC) Staining and Quantification

Five micrometer lung sections were incubated with the primary antibodies in 10% bovine serum in PBS overnight at 4°C. After washing with PBS buffer for 3 times, it was followed by the incubation with secondary and third antibodies for 1 h each at 37°C, respectively. Primary antibody dilutions used were as follows: α-SMA 1:500 and TβRII 1:200. The stained slides were mounted and visualized in bright-field with Olympus CX41 microscope. At least nine random microscopic fields were selected and the integral optical density (IOD) was calculated using Image-Pro Plus 6.0 software to determine the intensity of IHC staining.

### Gene Networks Analysis by IPA

Networks analysis of the targeted IPF related genes regulated by DLP and DHP were performed as described in our previous study (Lyu et al., 2017, 2018). The main source of disease targets for IPF was obtained from IPA^1^ database. According to our previous study (Dong J. et al., 2013), 15 compounds used as marker substances of DLP were uploaded into the IPA system to enable the discovery visualization, including gallic acid, puerarin, daidzin, peoniflorin, naringin, rosmarinic acid, salvianolic acid A, formononetin, calycosin, ethyl gallate, lipoteichoic acid, chlorogenic acid, ferulic acid, cryptotanshinone and tanshinone IIA. Similarly, 14 compound were chosen as major ingredients of DHP for IPA analysis, such as L-proline, L-phenylalanine, caffeic acid, danshensu, rutin, hydroxysafflor yellow A, safflor yellow A, salvianolic acid B, uridine, syringin, chlorogenic acid, ferulic acid, rosmarinic acid, salvianolic acid A (Liu et al., 2013). “Build-Path Explorer” module was applied to discover IPF-related targets, and the relationship between the targets and DLP or DHP ingredients, respectively.

### Statistical Analysis

All analyses were performed using SPSS 9.0 software (SPSS Inc., Chicago, IL, United States) or GraphPad (GraphPad Prism 6, San Diego, CA, United States). The results are expressed as the mean ± standard deviation. Multigroup comparisons of the means were carried out by one-way analysis of variance (ANOVA) test with *post hoc* Tukey’s test. The statistical significance for all tests was set at *P*-values of less than 0.05.

## Results

### DLP, but Not DHP, Protects Mice Against BLM-Induced Lung Injury

To evaluate the therapeutic effects of DLP and DHP in the bleomycin-induced lung fibrosis, we preformed serial μCT scans on mice before and after DLP, DHP, or ROS administration. Micro-CT imaging has been used as a diagnostic standard to classify the severity of lung fibrosis in patients in correlation to other pathologic findings (Lee et al., 2009; Jin et al., 2012).

**FIGURE 1:**
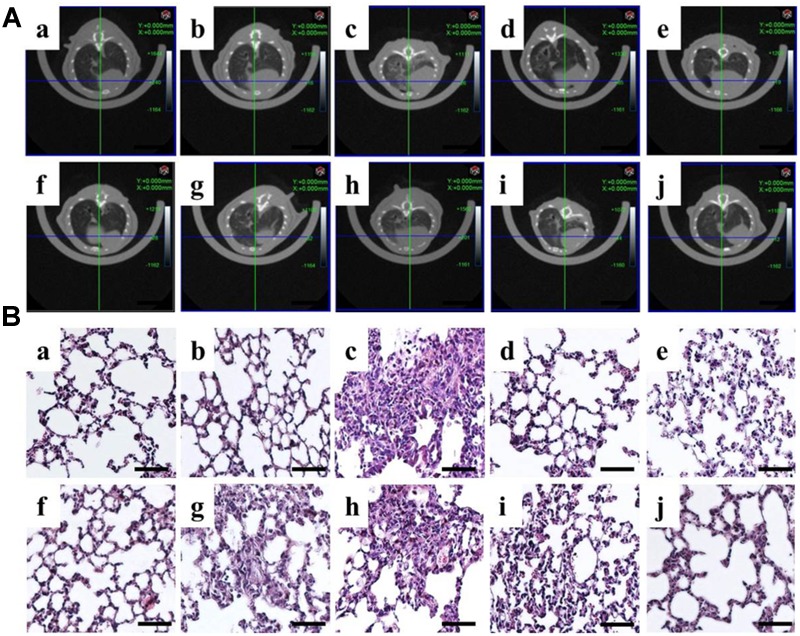
Correlation of *in vivo* μCT imaging with lung pathology in bleomycin-induced pulmonary fibrosis. **(A)**μCT scan and **(B)** H&E staining of mouse lungs from a, normal; b, sham; c, model; d, DLP low; e, DLP medium; f, DLP high; g, DHP low; h, DHP medium; i, DHP high; j, ROS. Scale bar = 50 μm.

Normal and sham groups (each group *n* = 6) did not reveal any lung abnormality from μCT imaging (Figure 1Aa,b). After the bleomycin treatment for 2 weeks, we chose 36 mice performing inflammation and fibrosis to test the effects of drugs. Different concentrations of DLP, DHP and ROS were administered to these 36 mice for 2 weeks at 24 h intervals, the μCT were analyzed before and after the treatments. No animals died during the entire treatment period. In contrast, in the model group without any drug treatments (*n* = 6), 4 mice died after 4 weeks and 2 mice were found with an increased attenuation with obscuration of the pulmonary vessels, which indicated the lung fibrosis (Figure 1Ac). The BLM-treated mice show great tolerance with higher concentration of DLP or DHP after 4 weeks treatments, even up three times of clinical dosage, 1590 mg/kg and 7.5 ml/kg separately. Drug treatment had no marked influence on the body weight of mice. Figure 1Ad indicated DLP with low concentration affect the inflammatory area, while high concentrations of DLP (≥530 mg/kg) sharply reduced the degree of BLM-induced fibrosis and reversed pathological damage to the high extent (Figure 1Ae,f). In contrast, mice treated with different concentrations of DHP (Figure 1Ag–i) show marginally effect on the pulmonary fibrosis. Positive control experiment shows that level of lung fibrosis treated with ROS were greatly reduced, as shown in Figure 1Aj.

HE stain further demonstrated histopathological changes in the form of severely damaged architecture and thickened interstitium in the lung tissues from BLM-treated mice, compared with those from control and sham mice (Figure 1Ba–c). These pathological changes were significantly alleviated by subsequent treatment with DLP (Figure 1Bd–f) or ROS (Figure 1Bj). In contrast, the DHP only marginally alleviated BLM-induced lung injury (Figure 1Bg–i). Thus, the tissue pathology findings correlated well with those of lung μCT imaging.

**FIGURE 2:**
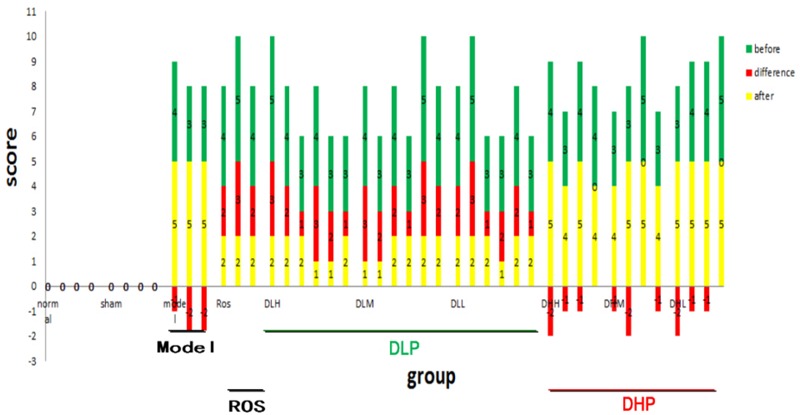
Quantification of the degrees of pulmonary fibrosis by μCT imaging before and after DLP, DHP, or ROS treatments. X axis represents individual animals in different treatment groups. Scores on Y axis represent severity level of BLM-induced fibrosis. Green and yellow bars indicate before and after drug treatment, respectively, and red bar indicates the differences in between. Negative value means no therapeutic effects.

Next, we quantified the degree of pulmonary fibrosis before (at 2 weeks) and after (at 4 weeks) drug-administration using a scale adapted from clinical practice (Lee et al., 2008; Figure 2). After DLP and ROS treatments, the average degree of fibrosis were reduced, where bronchial wall became thinner and reticular opacity was greatly reduced. Specifically, statistical analysis of the μCT lung fibrosis data (Table 1) indicated that the mean total score before and after ROS administration were 4.3 ± 0.5 and 2.0 ± 0, respectively (*p* = 0.0001). DLP treatment yielded a marked decrease from 3.7 ± 0.8 to 1.8 ± 0.4 (DLP low dose), from 3.8 ± 0.8 to 1.7 ± 0.5 (DLP medium dose) and from3.7 ± 0.8 to 1.7 ± 0.5 (DLP high dose), respectively, with *p*-value less than 0.003. In contrast, DHP treatment did not lead to any reduction in μCT scores (*p* > 0.05). Therefore, DLP but not DHP is effective in reducing fibrosis in the lung of BLM-induced mice.

**Table 1:** Comparison of μCT findings before and after administration of ROS, DLP or DHP on BLM-induced lung fibrosis in mice.

	Before	After	*p-*value
Normal	0	0	–
Sham	0	0	–
Model	3.3 ± 0.6	5.0 ± 0	0.0377
DLP low	3.7 ± 0.8	1.8 ± 0.4	0.0019
DLP medium	3.8 ± 0.8	1.7 ± 0.5	0.0009
DLP high	3.7 ± 0.8	1.7 ± 0.5	0.0028
DHP low	4.0 ± 0.8	5.0 ± 0	0.0917
DHP medium	3.5 ± 1.0	4.5 ± 0.6	0.0917
DHP high	3.8 ± 0.5	4.5 ± 0.6	0.0577
ROS	4.3 ± 0.5	2.0 ± 0	0.0001

### DLP Better Mitigates Pathological Changes Than DHP in Lung Tissues of BLM-Induced Fibrosis Mice

Next, we investigated whether these drugs could also mediate mitigation of pulmonary fibrosis by using Masson’s trichrome staining. Lung sections were selected and stained by Masson. BLM-treated mice have been revealed severe pulmonary fibrosis comparing with normal slides. Followed series of concentrations of DLP, DHP and ROS were added to the mice model by intragastric administration, we observed the change of collagen deposition areas.

**FIGURE 3:**
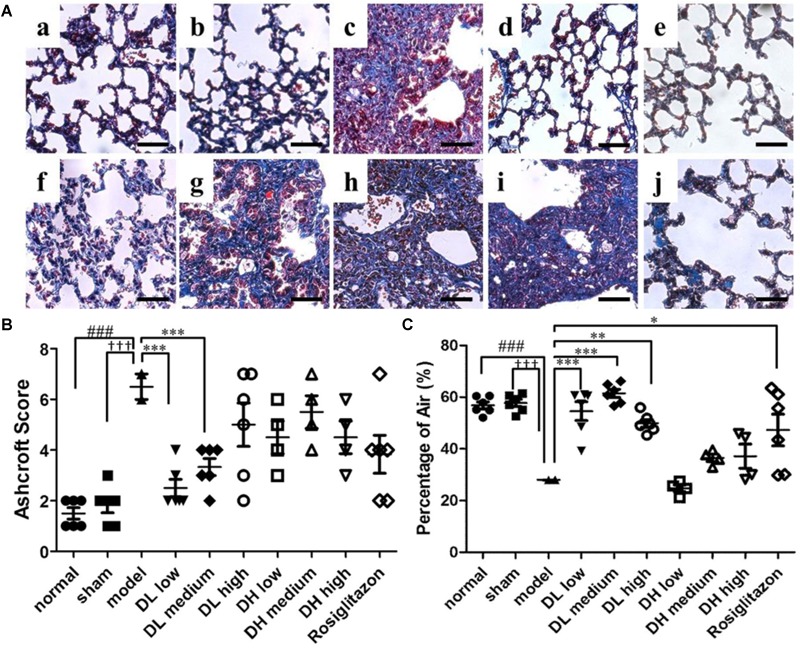
Effects of DLP and DHP on pulmonary fibrosis in BLM-induced mice model. **(A)** Representative images of Masson’s trichrome staining results, scale bar = 50 μm. Five micrometer sections of lung central area with different treatment were stained with Masson. a, normal; b, sham; c, model; d, DL low dose; e, DL medium dose; f, DL high dose; g, DH low dose; h, DH medium dose; i, DH high dose; j, ROS. **(B)** Comparison of the Ashcroft score among the experimental groups. **(C)** Comparison of the alveolar air area among the experimental groups. Results are expressed as the mean ± standard deviation. ^###^*P* < 0.001 versus the normal group; ^†††^*P* < 0.001 versus the sham group; ^∗∗∗^*P* < 0.001, ^∗∗^*P* < 0.01 and ^∗^*P* < 0.05 versus the model group.

Normal, sham, model-operated mice section slides were shown in Figure 3Aa–c. Figure 3Ad–f show that all the tested dosages of DLP had markedly reduced the area of collagen deposition in pulmonary fibrosis. While DHP didn’t cause any decrease of the pulmonary lesion in BLM-treated mice (Figure 3Ag–i). In addition, the Ashcroft score was then used to determine the area of lung fibrosis. The data indicated that, following low- and medium-dose DLP administrations, the scores of mice with pulmonary fibrosis were significantly decreased, when compared with that of the model group, while DHP administrations were not statistically significant, when compared with that of the model group (Figure 3B). Fully in agreement with these results, the percentage of alveolar air area was significantly increased following low-, medium- and high-DLP administrations in bleomycin-treated mice compared with that of the model group, while DHP administrations were not statistically significant, when compared with that of the model group (Figure 3C).

These findings indicated an anti-fibrotic effect of DLP on pulmonary fibrosis in mice. One of the possible explanations could be that DLP has better expectorant effects than DHP, due to pulmonary fibrosis leading to sputum in the lung, so that DLP has stronger therapeutic effects on pulmonary fibrosis than DHP.

### DLP, but Not DHP, Inhibits Fibroblast Differentiation Into Myofibroblasts

**FIGURE 4:**
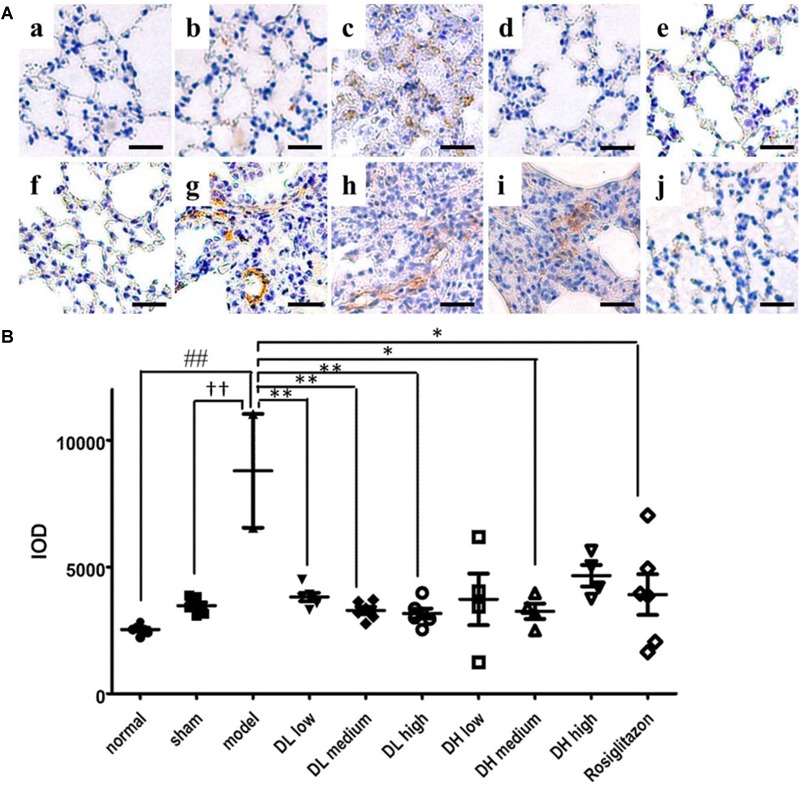
DLP reduces BLM induced upregulation in pulmonary α-SMA expression in mice. **(A)** Representative images of immunohistochemical staining for α-SMA protein in lung tissues. Scale bar = 50 μm. Panel indicator a, normal; b, sham; c, model; d, DL low dose; e, DL medium dose; f, DL high dose; g, DH low dose; h, DH medium dose; i, DH high dose; j, ROS. **(B)** Integral optical density of α-SMA positive expression in lung tissues. IOD: integral optical density. Results are expressed as the mean ± standard deviation. ^##^*P* < 0.01 versus the normal group; ^††^*P* < 0.01 versus the sham group; ^∗∗^*P* < 0.01 and ^∗^*P* < 0.05 versus the model group.

Myofibroblasts are the primary cell type involved in ECM synthesis and tissue remodeling, which leads to the loss of alveolar function (Scotton and Chambers, 2007). The hallmark of its differentiation is the expression of α-SMA. As shown in Figure 4Ac, mice treated with BLM exhibited severe lung fibrosis, where α-SMA expression sharply increased, as indicated by the intensity of its labeled antibody (brown). In comparison, mice in sham or normal groups showed few α-SMA expression and without any inflammation (Figure 4Aa,b). These immunohistochemistry results indicated that pulmonary α-SMA protein expression increased following BLM instillation, but significantly decreased by the following low, medium and high dose-DLP treatment (Figure 4Ad–f). In addition, Figure 4B showed the integral optical density of α-SMA positive expression in lung tissue samples, indicating only medium dose-DHP could significantly down-regulated the α-SMA protein expression, neither low-dose nor high-dose DHP could reduce the expression level of α-SMA in BLM-induced lung tissues (Figures 4Ag–j).

### DLP Down-Regulates the Expression of TGF-β Receptor II More Efficiently Than DHP in BLM-Induced Lung Fibrosis

**FIGURE 5:**
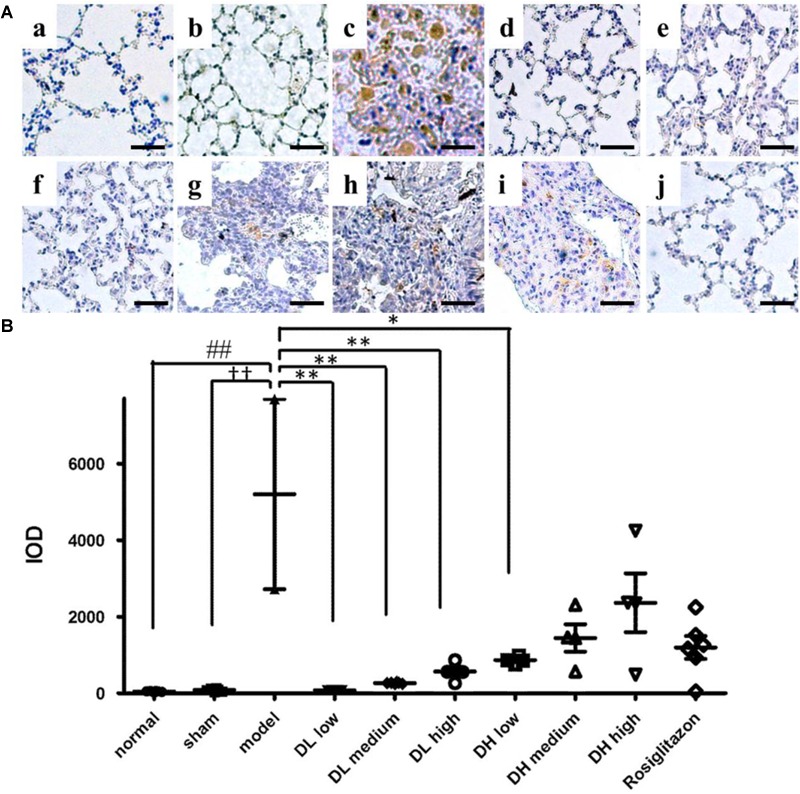
Effects of DLP and DHP on TGF-β receptor type II expression in lung tissues of mice. **(A)** Representative images of immunohistochemical staining for TβRII protein in lung tissue samples. Scale bar = 50 μm. Panel indicators: a, normal; b, sham; c, model; d, DL low dose; e, DL medium dose; f, DL high dose; g, DH low dose; h, DH medium dose; i, DH high dose; j, ROS. **(B)** Integral optical density of TβRII-positive expression in lung tissue samples. IOD: integral optical density. Results are expressed as the mean ± standard deviation. ^##^*P* <0.01 versus the normal group; ^††^*P* < 0.01 versus the sham group; ^∗∗^*P* < 0.01 and ^∗^*P* < 0.05 versus the model group.

Since the epithelium-specific deletion of TGF-β receptor type II (TβRII) plays a critical role in protecting mice from bleomycin-induced pulmonary fibrosis (Li et al., 2011), we examined the effects of DLP and DHP on the expression levels of TβRII by immunohistochemistry staining and image analysis. As depicted in Figure 5Ac, BLM instillation resulted in significant increase of TβRII expression in lung tissues of mice. After 2 weeks of drug treatments, mice treated with low, medium and high dose-DLP all showed the expression levels of TβRII receptors reduced significantly, as Figure 5A,B *P*-value indicated. In contrast, only low dose-DHP administration could significantly down-regulated the TβRII expression level, while the TβRII expression levels after medium and high dose-DHP treatments showed slightly reduced while the differences were shown no statistically significant.

### Analysis of the Ingredient-Target-Disease Network of DLP and DHP

**FIGURE 6:**
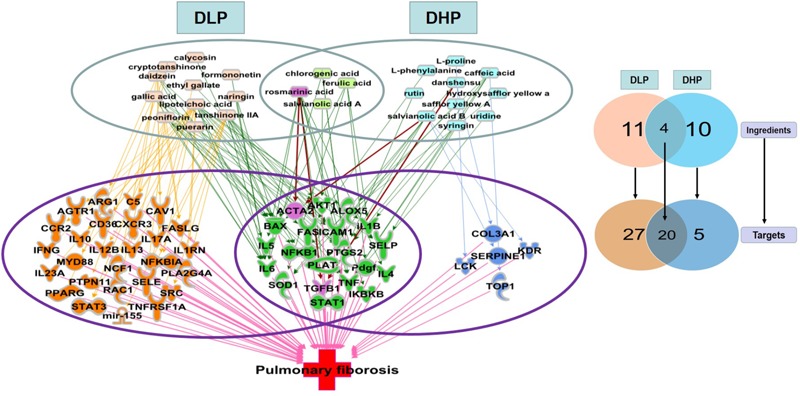
Unique and shared pulmonary fibrosis-related targets regulated by DLP and DHP. Fifteen DLP major ingredients (pink), 14 DHP major ingredients (blue), and four shared ingredients from these two prescriptions (light green) were represented, which cooperatively modulate 52 common intracellular targets. Among these 52 pulmonary fibrosis-related targets, 27 unique targets regulated by DLP (orange) and 5 unique targets regulated by DHP (dark blue) with 20 targets shared by both (green).

To explain the underlying mechanism that contributed to the differential efficacy of DLP and DHP for pulmonary fibrosis, we identified the unique and shared pulmonary fibrosis related targets of DLP and DHP by IPA. According to the qualitative and quantitative analysis of the major ingredients in DLP and DHP in our previous studies (Dong J. et al., 2013; Liu et al., 2013), 15 potential active ingredients of DLP and 14 potential active ingredients of DHP were used to establish the ingredient-target-disease network. These 25 ingredients cooperatively modulate 52 common pulmonary fibrosis related intracellular targets, among which 27 were uniquely regulated by DLP, five were uniquely regulated by DHP and 20 were shared by both DLP and DHP (Figure 6).

Consistent with our previous results of immunohistochemical staining, ACTA2 (encoding α-SMA) was shown to be regulated by both DLP and DHP. DHP mainly regulated pulmonary fibrosis-related inflammatory genes and signaling pathways, such as IL1β, IL4, IL5, IL6, TGFβ1, and TNFα. In addition to the above inflammatory related targets, IL10, IL17A, IL23A, ARG1, and AKT1 were also regulated by DLP ingredients. It’s worth noting that DLP could regulate not only inflammation related genes and signaling pathways, but also myofibroblast differentiation and collagen secretion related genes, such as PTPN11, RAC1, PPARG. It suggested that DLP inhibited pulmonary fibrosis through suppressing both pro-inflammatory and pro-fibrotic pathways.

## Discussion

Pulmonary fibrosis is a devastating lung disorder with mysterious pathogenesis and limited treatment options. Although several drug treatment options are available, most of them are neither suitable for clinical use nor cannot be safely applied in targeted therapy. Findings from published placebo-controlled trials in IPF have established that pirfenidone and nintedanib prevent about 50% of the decline in forced vital capacity typically seen in this disease (King et al., 2014; Richeldi et al., 2014; Wuyts et al., 2014). Moreover, statins and dexamethasone were reported recently that may have a beneficial clinical outcomes in IPF, while it may also be associated with the presence of interstitial lung abnormalities (Shi et al., 2014; Kreuter et al., 2016). It seems that targeted therapies are unlikely to work well in isolation since multiple co-activated pathways are involved in the pathogenesis of IPF. To date, combination therapy becomes an attractive toolkit to deal with diagnostic uncertainty and to suppress both pro-inflammatory and pro-fibrotic pathways at the same time. Thus, many Chinese herbal medicines, as the combination medicines present their advantages of exerting anti-inflammatory effects and removing fibrosis. Previously, Gualou Xiebai Baijiu Decoction (GXD) has been reported to prevent myocardial fibrosis by blocking TGF-β/Smad signaling in rats (Ding et al., 2013). GXD consists of Trichosanthes kirilowii Maxim and Allium macrostemon Bge in a weight ratio of 2:1. Paeoniflorin, as one typical TCM was tested in liver fibrosis and proved to affect HIF-1α through mTOR-dependent pathway (Zhao et al., 2014). Salvia miltiorrhiza Bge. (Danshen) could ameliorate hypertrophy and dilatation of renal tubule and glomeruli possibly by decreasing the expression of collagen and fibronectin in association with suppression of TGF-β1/Smad pathway (Xu et al., 2016). One of its active component, Tanshinone IIA, was shown to alleviate the fibrosis in TGF-β1-induced murine fibroblast cells and attenuate BLM-induced pulmonary fibrosis (He et al., 2015).

The intratracheal instillation of bleomycin in mice induces early damage to alveolar epithelial cells and development of inflammation followed by fibrotic tissue changes and represents the most widely used model of pulmonary fibrosis to investigate human IPF. Although several earlier studies used this model to explore natural product or herbal derived compounds for the treatment of IPF, our study was the first to integrate μCT and histological analyses for compound Chinese medicine prescription. Our μCT data showing the extent (%) of fibrosis correlated with histological read-outs in Ashcroft score and the percentage of alveolar air area and was able to quantify effectively and non-invasively disease progression longitudinally and to reduce the variability and number of animals used to assess the damage.

It is well-recognized that the uncontrolled proliferation of lung fibroblasts and differentiation of fibroblasts into myofibroblasts excessively produce extracellular matrix (ECM) proteins which contribute to the fibrosis change of the lungs. Here, guided by clinical observations and TCM theory for “removing both phlegm and blood stasis,” we proposed that DLP, a proven cardiovascular Chinese medicine prescription could be effective in treating fibrotic pulmonary diseases. Physiological parameters, pathological histology, and μCT imaging were used to quantify the lung function, degree of pulmonary fibrosis before and after the medicine treatments. In addition, immunohistochemistry staining and image analysis were used to investigate the effects of DLP and DHP on the expression levels of α-SMA and TβRII. Our results revealed that the expression levels of α-SMA and TβRII all reduced significantly after treated by low, medium and high dose-DLP compared with that of the model group in lung tissues of mice. In contrast, DHP could not significantly down-regulate the expression levels of α-SMA and TβRII simultaneously, whatever in low, medium or high dose. Based on the results, it was hypothesized that DLP, instead of DHP can better mitigate BLM-induced pulmonary fibrosis by inhibiting the TGF-β signaling activated myofibroblast differentiation and α-SMA expression in a mouse model. Although the core analysis by IPA revealed that DLP ingredients regulated a number of pulmonary fibrosis related inflammatory genes, such as IL1β, IL4, IL4, IL6, IL12B, IL13, IL17A, IL10, IL23A, TNFα, ARG1, and AKT1, the effect of DLP on restraining inflammatory responses induced by BLM at the early phase of IPF still needs further investigation. It was reported that IL10, ARG1, and AKT1 were all associated with the macrophage activation and apoptosis in pulmonary fibrosis. IL-10 could induce lung fibrosis via promoting fibrocyte recruitment and M2 macrophage activation in a CCL2/CCR2 axis (Sun et al., 2011). ARG1 could regulate the macrophage phenotype transition (Yao et al., 2018) and Akt1-mediated mitophagy contributed to alveolar macrophage apoptosis resistance and was required for pulmonary fibrosis development (Larson-Casey et al., 2016). In addition, DLP could regulate not only inflammation related genes and alveolar macrophage activation, but also genes associated with myofibroblast differentiation and collagen secretion, such as PTPN11, RAC1, PPARG. PTPN11 (coding SHP2) was an important regulator of fibroblast differentiation, and loss of SHP2 expression or activity was sufficient to induce fibroblast-to-myofibroblast differentiation in primary human lung fibroblasts (Tzouvelekis et al., 2017). PPARG is also an important regulator of fibroblast/myofibroblast activation and suggest a role for PPARG ligands as novel therapeutic agents for fibrotic lung diseases (Milam et al., 2008). Rac1 could inhibit collagen deposition through regulating matrix metalloproteinases-9 (MMP-9) transcription (Murthy et al., 2010). In addition, IL-17 directly promoted the proliferation, transformation and collagen synthesis of lung fibroblasts via the NF-kB signaling pathway (Dong Z. et al., 2013). MiR-155 was also a potential target of DLP for the treatment of pulmonary fibrosis, which was a critical miRNA that drived fibrosis and was required for inflammasome-mediated collagen synthesis during fibrosis (Artlett et al., 2017).

It is also worth noting that although both of DLP and DHP contain the Chinese herbal Salvia miltiorrhiza Bge. (Danshen), different dosage forms may lead to different content of active ingredients. It has been reported that the amount of active ingredient required for transdermal delivery can be significantly less than that for oral systems. Furthermore, different drug delivery systems will cause the differences in drug stability and drug concentration of target organs, which may lead to different pharmacodynamic effects. In the future, our study will focus on investigating the active components that inhibit the pulmonary fibrosis in DLP.

To the best of our knowledge, this is the first study to prove the therapeutic effect of an approved cardiovascular medicine, DLP in pulmonary fibrosis, with combination of fibrosis reduced scores levels. Furthermore, we demonstrate the possible mechanism of DLP pathway and explained the difference between DLP and DHP. Comparing to DHP, DLP is not only a “blood stasis syndrome” medicine, it also has functions of “removing both phlegm and blood stasis” in suppressing both pro-inflammatory and pro-fibrotic pathways. It suggests a successfully repurposed of a clinically efficacious cardiovascular Chinese medicine can be treated previously unknown pulmonary fibrosis based on TCM theory.

## Ethics Statement

This study was carried out in accordance with the recommendations of Guidelines for Animal Experiments of Tianjin University of Traditional Chinese Medicine. The protocol was approved by the Institutional Animal Care and Use Committee at the Tianjin International Joint Academy of Biotechnology and Medicine (TJAB-JY-2011-002).

## Author Contributions

RS performed the research, analyzed the data, and wrote the manuscript. FW contributed to animal experiments and μCT image analysis. ML contirbuted to IPA analysis. JY revised the manuscript. PZ contributed to preparation of Danlou prescription and Danhong prescription. YZ designed and funded the research, interpreted the data, and finally approved the submission of this manuscript.

## Conflict of Interest Statement

The authors declare that the research was conducted in the absence of any commercial or financial relationships that could be construed as a potential conflict of interest.

## Footnotes

1 http://www.ingenuity.com
